# Prognostic risk model development and prospective validation among patients with cervical cancer stage IB2 to IIB submitted to neoadjuvant chemotherapy

**DOI:** 10.1038/srep27568

**Published:** 2016-06-09

**Authors:** Kecheng Huang, Haiying Sun, Xiong Li, Ting Hu, Ru Yang, ShaoShuai Wang, Yao Jia, Zhilan Chen, Fangxu Tang, Jian Shen, Xiaomin Qin, Hang Zhou, Runfeng Yang, Juan Gui, Lin Wang, Xiaolin Zhao, Jincheng Zhang, Jiong Liu, Lili Guo, Shuang Li, Shixuan Wang

**Affiliations:** 1 From Department of Obstetrics and Gynecology, Tongji Hospital, Tongji Medical College, Huazhong University of Science and Technology, Wuhan, Hubei, China; 2 Department of Obstetrics and Gynecology, Wuhan Central Hospital, Wuhan, Hubei, China; 3 Henan Cancer Hospital, Zhengzhou, Henan, China; 4 Department of Obstetrics and Gynecology, Wuhan General Hospital of Guangzhou Military Command, Wuhan, Hubei, China; 5 Department of Obstetrics and Gynecology, Xiangfan Central Hospital, Tongji Medical College, Huazhong University of Science and Technology, Xiangfan, Hubei, China; 6 Department of Obstetrics and Gynecology, Nanjing Drum Tower Hospital, the Affiliated Hospital of Nanjing University Medical School, Nanjing, Jiangsu, China; 7 Hubei Tumor Hospital, Wuhan, Hubei, China; 8 Renmin Hospital, Wuhan University, Wuhan, Hubei, China; 9 Tai-He Hospital, Hubei University of Medicine, Shiyan, Hubei, China; 10 Shanghai Jiao Tong University School of Medicine, Shanghai, China

## Abstract

This study was designed to develop a risk model for disease recurrence among cervical cancer patients who underwent neoadjuvant chemotherapy and radical surgery. Data for 853 patients were obtained from a retrospective study and used to train the model, and then data for 447 patients from a prospective cohort study were employed to validate the model. The Cox regression model was used for calculating the coefficients of the risk factors. According to risk scores, patients were classified into high-, intermediate-, and low-risk groups. There were 49 (49/144, 34%) recurrences observed in the high-risk group (with a risk score ≥ 2.65), compared with 3 (3/142, 2%) recurrences in the low-risk group (with a risk score < 0.90). Disease-free survival (DFS) was significantly different (log-rank *p* < 0.001) among the three risk groups; the risk model also revealed a significant increase in the accuracy of predicting 5-year DFS with the area under the ROC curve (AUC = 0.754 for risk model vs 0.679 for FIGO stage system); the risk model was also validated with data from the prospective study (log-rank *p* < 0.001, AUC = 0.766). Both high-risk and intermediate-risk patients can be more effectively identified by this risk model.

Cervical cancer is the second most commonly diagnosed malignant tumour in undeveloped areas^1^. In addition to traditional therapy, other therapies have also been used for the medical care of patients with cervical cancer. One of these therapies is neoadjuvant chemotherapy (NACT) plus surgery together with adjuvant post-surgical therapy, which may represent a hopeful alternative to chemoradiation. Several studies have also examined this innovation^2^,^3^,^4^,^5^,^6^, including randomized clinical trials, prospective cohort studies and case-control studies worldwide^2^,^7^,^8^,^9^,^10^,^11^,^12^,^13^,^14^,^15^,^16^. Why has NACT been so widely investigated? Several reasons should be considered. First, precision radiotherapy units are rare in undeveloped areas, such as certain parts of China; thus, clinicians must resort to neoadjuvant chemotherapy to shrink tumours to perform surgery^10^,^11^,^12^,^13^,^17^,^18^. Second, studies have shown significant increases in survival after subjection to NACT compared with primary radiotherapy or surgery^8^,^19^. Third, traditional treatment for cervical cancer consists of radical surgery or radiotherapy without sparing fertility, which leads to psychosexual dysfunction and decreased quality of life. However, NACT allows clinical and pathological assessments of a tumour’s response to a particular chemotherapeutic regimen and hence provides an opportunity to optimize therapy, including fertility-preserving therapy^20^,^21^. As a result, a new era for locally advanced cervical cancer (LACC) has begun in recent years due to the facility of NACT. However, few risk models with prospective validation have been performed for NACT patients. Consequently, evaluations of recurrence rates are less accurate, as is identification of patients at high-risk for recurrence.

This study was designed to define a risk model for predicting the probability of recurrence using data from a retrospective study that included 853 NACT patients; then, the risk model was validated using data from a prospective cohort. Using these data, new conclusions can be drawn regarding patients with the greatest recurrence rates. Moreover, this model can be used to select patients to participate in new management protocols or for future research, and it may also provide measures to prevent disease recurrence.

## Results

A total of 1300 patients were examined during the study (data for 853 patients were obtained from the retrospective study and were used as training data to define the new risk model, and data for 447 patients were obtained from a prospective cohort for validation of the risk model). Patient characteristics for both datasets are shown in Table 1. A chart depicting the flow of patients in the prospective cohort is shown in Supplementary Fig. S1.

### Risk factor selection

Univariate analysis and Cox regression were used to identify promising risk factors. Clinical risk factors, such as age, FIGO stage, and tumour size, were evaluated, as well as pathological risk factors, including grade, cell type, lymph vascular space invasion (LVSI), parametrial infiltration, vaginal surgical margin and lymph node metastasis. The results of the univariate analysis are shown in Table 2; age, FIGO stage, grade, cell type, parametrial infiltration and lymph node metastasis were significantly associated with disease free survival (*p* < 0.05).

### Risk model training

We added risk factors with *p* < 0.05 to the multivariate Cox regression model. Data in Table 3 show that FIGO stage, grade, cell type, parametrial infiltration and lymph node metastasis were independent prognostic predictors of disease-free survival (DFS).

Then, a risk score was calculated from the final multivariate Cox regression model by incorporating the six risk factors, which were weighted by their Cox regression coefficients with adjustment of β0 = 0.22. The quartiles (Q1, Q3) of risk score were 0.90 and 2.65, respectively.

Accordingly, the risk score was further split into three groups: a low risk group (risk score < 0.90), an intermediate risk group (0.90≤ risk score < 2.65), and a high risk group (risk score ≥ 2.65). A total 142 patients were classified into the low risk group, with 3 (2.1%) recurrences, and 144 patients were classified into the high risk group, with 49 (34.0%) recurrences.

**Figure 1:**
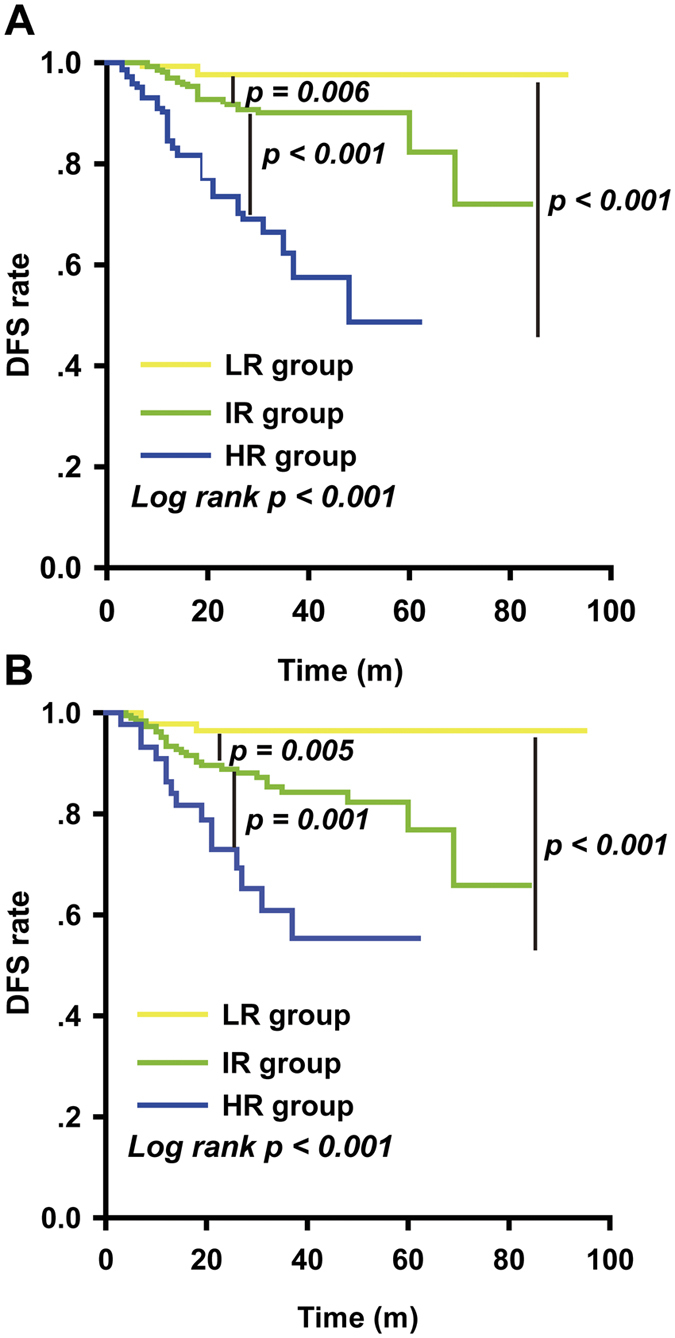
Kaplan-Meier survival estimates of evaluated patients with cervical cancer from both the (**A**) training study and the (**B**) validation cohort. Kaplan-Meier survival estimates for low-, intermediate- and high-risk patients with cervical cancer, as defined by the risk model. Disease-specific survival curves of evaluated patients in the (**A**) training study and (**B**) validation cohort. Log-rank test was used to calculate *p* values. Statistical significance was observed between the groups.

A log-rank test was also used to assess the recurrence rates between the risk groups. Figure 1A and Table 4 show that the high-risk group had the lowest 5-year DFS, at 48.7%, while the low-risk group had the highest 5-year DFS, at 97.7%. The differences between groups were statistically significant (low risk group vs intermediate risk group, *p* = 0.006; high risk group vs intermediate risk group, *p* < 0.001) (Fig. 1A). The FIGO stage system was also used to predict recurrence rates (Supplementary Fig. S2A online). Although the three groups were also classified with log-rank *p* = 0.002, the difference between stages IB2 and IIA was not significant (*p* = 0.07), nor was the difference between stages IIA and IIB (*p* = 0.21).

### Risk model validation

Data from a prospective cohort were used to evaluate the performance of the predictive model. Ninety patients were classified into the low-risk group, with 3 (3.3%) recurrences, and 44 patients were classified into the high-risk group, with 15 (34.1%) recurrences. Table 4 shows that the high-risk group had the lowest 5-year DFS, at 55.3%, while the low-risk group had the highest 5-year DFS, at 96.5%.

A log-rank test showed a statistically significant difference for DFS between groups, with *p* < 0.001 (low risk group vs intermediate risk group, *p* = 0.005; high risk group vs intermediate risk group, *p* = 0.001) (Fig. 1B). In the validation data, the difference between the IB2 and IIA groups was not statistically significant, not was the difference between stages IIA and IIB (IB2 vs IIA, *p* = 0.43; IIA vs IIB, *p* = 0.32). More details can be seen in Supplementary Fig. S2B online.

### Risk model assessment

**Figure 2:**
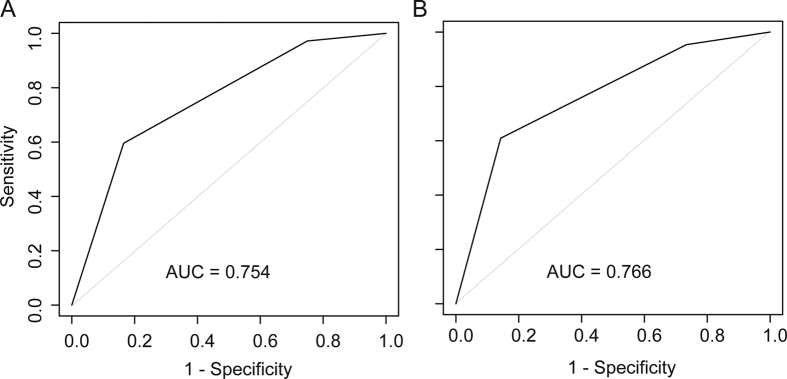
Time-dependent receiver operating characteristic (ROC) curves of evaluated patients with cervical cancer from both the (**A**) training study and the (**B**) validation cohort. ROC curves for the risk model were used as predictors of recurrence as result of cervical cancer within 5 years in the (**A**) training study and (**B**) validation cohort. The areas under the ROC curves were >0.75 in both the training study and the validation cohort.

A time-dependent ROC curve was employed to evaluate the discrimination of the risk model, a method used in a previous study^22^. Response was defined when recurrence occurred. The predictor for the ROC was group classification. The risk model in the training data had an AUC of 0.754 in predicting 5-year DFS, while the risk model in the validation data had an AUC of 0.766 (Fig. 2). In contrast, FIGO stage in the training data had an AUC of 0.679, while FIGO stage in the validation data had an AUC of 0.659 (Supplementary Fig. S3 online).

## Discussion

In recent decades, NACT has been applied as a new therapeutic approach for LACC because of unsatisfactory results with conventional therapy^8^,^19^,^23^,^24^,^25^. Our risk model with validation is a novel method that provides scientific clues to guide doctors in differentiating high risk cervical cancer patients from low risk cervical cancer patients; consequently, tailored therapy can be applied efficiently.

The risk model divided patients into three groups with high efficiency as significantly different recurrence rates among the groups were revealed by log-rank tests in both the retrospective training data and the prospective validation data. Moreover, the risk models showed superior predictability compared with the FIGO stage system. When time-dependent ROC curves were used to evaluate the discrimination of the risk model, the risk models also revealed high accuracy in predicting 5-year DFS. Compared with the FIGO stage system, the AUC of the risk model was much larger when the evaluation was performed with the training data and validation data.

Variables including FIGO stage, grade, cell type, parametrial infiltration and positive lymph node metastasis were demonstrated to be independent risk factors in multivariate Cox regression for DFS. LVSI and vaginal surgical margin were reported to be independent prognostic factors^13^,^26^,^27^, and these risk factors were statistically significant in univariate Cox regression for DFS during risk model training. Although these two margins were ultimately excluded from the multivariate Cox model, our study did validate previous findings^17^. The exclusion was partly because we adopted a strict entering standard when the risk model was defined using training data, and consequently, risk factors such as vaginal surgical margin and LVSI could not be included in the model. However, the FIGO stage system is still an important prognostic factor, as shown by previous studies^28^.

Presently, NACT has given scholars and patients new innovations for treating cervical cancer^7^,^20^,^21^, although chemo-radiation has also been an effective treatment for cervical cancer. Some scholars believe that there is a crucial need for randomized studies comparing NACT with chemoradiation^7^. Additionally, a distinguished trial aiming to solve this problem, which fascinates us greatly, was designed by the European Organization for Research and Treatment of Cancer (EORTC 55994). This promises a new standard with clearer instructions for cervical cancer treatment and will guide treatment for LACC based on the comparison results of this study. There have always been disputes regarding the optimal treatment for cervical cancer; thus, Professor Eddy conducted a randomized controlled trial to determine if NACT, along with radical hysterectomy and pelvic/para-aortic lymphadenectomy (RHPPL), could improve survival in stage IB cervical cancer. He found no evidence that NACT offered any benefit to patients undergoing RHPPL for bulky stage IB cervical cancer^29^. However, a meta-analysis consisting of 1071 patients from several trials showed a significant increase in survival in favour of NACT with surgery compared with surgery alone^19^. Furthermore, a meta-analysis of 21 randomized trials dating back to the early 1990s collected information regarding individual patients with LACC receiving NACT^23^. This study of 872 patients included a comparison of NACT with surgery (with or without radiotherapy) with radical radiotherapy alone. The comparison showed that women treated with NACT had a statistically significant reduction in risk of death (*p* = 0.0004). The protocol for this large meta-analytic study was completed in 1999, when radical radiation therapy was still the standard treatment for cervical cancer patients.

There are some limitations to this study. Our risk model is less predictive than the model in the previous study, which revealed a much larger AUC of >0.80^30^; this is partly because a new mathematical method of LASSO penalized the Cox regression used in their study, although it resulted in a more fitting Cox model. In the future, we should use this new mathematical method in our study to define a more fitting risk model. However, biomarkers, which may impact a malignant tumour’s progression^31^, were not included in our study; thus, in the future we will assess the biomarkers of cervical cancer to identify prognostic risk factors and to improve the predictability of the risk model.

In summary, a risk model based on a risk score was developed in this study. The risk model displayed good discrimination for 5-year DFS in both the training data and the prospective validation data. Three groups, including a high-risk group, an intermediate-risk group and a low-risk group, were categorized by this risk model. The highest recurrence rates were observed in patients in the high-risk group, who, in particular, need timely medical care and close follow-up. This risk model can be utilized to discriminate low-risk patients from intermediate or high risk patients, who may benefit from new or different therapeutic approaches. This may help save time and money for patients and make it easier for clinicians to identify high-risk patients. The risk model provides some clues to clinicians for preventing the recurrence of cervical cancer, with important cost-efficiency implications.

## Methods

### Eligibility

The study was carried out in accordance with approved guidelines. The retrospective data of patients in Central China was used for the training data. Data from patients in the prospective cohort were used for validation. The information for the retrospective study dated from 1999, and the follow-up lasted until 2008. The cohort was performed at the Departments of Obstetrics and Gynaecology at our institutions, and the registration number at Clinicaltrials.gov was NCT01628757. Sixteen institutions have taken part in the study, and eligible patients were diagnosed with cervical cancer by pathological experts according to cervical biopsy and staged as IB2 to IIB by clinicians, according to the International Federation of Gynecology and Obstetrics (FIGO). The inclusion criteria included age ≥ 18, measurable lesions and the possibility of undergoing radical surgery, as well as a Karnofsky Performance Status ≥70, normal EKG, normal chest X-ray, normal blood cell count, normal hepatic function, and normal renal function. The exclusion criteria included preexisting sensory or motor neuropathy greater than WHO grade 1 or history of myocardial infarction or cardiac insufficiency ≥grade 3 (New York Heart Association scale); patients previously treated for cervical cancer (e.g., surgery, chemotherapy or radiotherapy) and patients with a previous or current history of other neoplasms, as well as patients with active infectious diseases or other medically complicating conditions, were excluded. Pregnant or lactating women were also excluded from this study.

The clinical investigation followed the Declaration of Helsinki and was carried out in accordance with approved guidelines. All experimental protocols were approved by the Ethics Committee of Tongji Medical College at Huazhong University of Science and Technology. All eligible patients gave written informed consent before entering this study.

### Evaluation of short-term response

The clinical response of bidimensionally measurable and assessable disease was classified as complete response (CR), partial response (PR), stable disease (SD) or progressive disease (PD), which was the same classification that was adopted in previous research, according to WHO criteria^7^,^17^.

After completion of the safety follow-up (i.e., 28 days after surgery), the decision regarding systemic adjuvant therapy was made by the treating physician. Patients who had traditional risk factors, such as vascular involvement, parametrial extension, deep stromal invasion, positive lymph nodes, or a positive surgical margin, received postoperative adjuvant therapy, such as irradiation or chemotherapy.

### Follow-up study

DFS was defined as the time from the first day of assignment until the date of first relapse or death (regardless of cause)^7^,^32^. Monitoring comprised a pelvic physical examination and vaginal cytology examination; magnetic resonance imaging (MRI) or computed tomography (CT) of the pelvic cavity and abdomen, as well as a chest x-ray, was carried out every 6 months for the first 2 years and once a year thereafter.

### Statistical analysis

Both the univariate and multivariate Cox model were used. Three steps were performed to train and validate the risk model for recurrence according to the principles proposed by previous studies^30^,^33^. **Step I – Predictive risk factor selection.** Univariate Cox regression analyses were performed to screen out potential risk factors by estimating the hazard ratios (HR) together with their 95% confidence intervals (CIs); factors with *p* < 0.05 were retained for use in step II. **Step II – Risk model training.** Multivariate Cox regression was carried out for construction of the risk model. Meanwhile, predictive risk factors were automatically retained by the computer if a factor had a *p* < 0.05 or was necessary. Then, the effects of potential predictive risk factors were assessed using a risk score analysis based on a linear combination of the selected risk factors weighted by the Cox regression coefficient. **Step III – Risk model validation.** Data from a prospective cohort study were used to evaluate the risk model’s performance in predicting high-risk patients and low-risk patients. The time-independent ROC curve was employed to evaluate the discrimination of the risk model and the FIGO stage system, which was performed by R software. As described by previous studies, the model is considered to have good discrimination if the area under the ROC curve (AUC) is greater than 0.75^34^,^35^. All *p*-values were two-tailed, and values < 0.05 were considered statistically significant. All statistical analyses were carried out using the IBM SPSS 20.0 statistical software package.

## Additional Information

**How to cite this article**: Huang, K. *et al.* Prognostic risk model development and prospective validation among patients with cervical cancer stage IB2 to IIB submitted to neoadjuvant chemotherapy. *Sci. Rep.* **6**, 27568; doi: 10.1038/srep27568 (2016).

## Supplementary Material

Supplementary Information

## Figures and Tables

**Table 1:** Clinical characteristics for all patients.

Characteristic	training (n = 853)		validation (n = 447)	
	No.	%	No.	%
Age(25th-75th percentiles) (year)				
Median	44		45	
Range	39–50		40–49	
Tumor size(25th-75th percentiles) (cm)				
Median	4.0		4.0	
Range	3.5–5.0		3.0–5.0	
Tumor grade				
G1	58	6.8	35	7.8
G2	354	41.5	216	48.3
G3	240	28.1	157	35.1
Undetermined	201	23.6	39	8.7
FIGO stage				
IB2	220	25.8	124	27.7
IIA	265	31.1	112	25.1
IIB	368	43.1	211	47.2
Cell type				
Squamous	756	88.6	393	87.9
Non-squamous	91	10.7	51	11.4
Unknown	6	0.7	3	0.7

FIGO, International Federation of Gynecology and Obstetrics.

**Table 2:** Univariate Cox regression for DFS.

Variables		β*	HR	P
Age	>44 vs ≤44 years	0.48	1.61	0.03
FIGO stage	IIA vs IB2	0.78	2.18	0.02
	IIB vs IB2	0.89	2.44	0.004
Tumor size	>4 cm vs ≤4 cm	0.31	1.37	0.19
Grade	G2 vs G1	0.77	2.16	0.20
	G3 vs G1	1.22	3.38	0.04
	Undetermined vs G1	0.72	2.05	0.25
Cell type	Squamous vs non-squamous	0.81	2.24	0.003
LVSI	Positive vs negative	0.34	1.40	0.29
Parametrial infiltration	Positive vs negative	0.96	2.61	<0.001
Vaginal surgical margin	Positive vs negative	0.65	1.91	0.13
Lymph node metastasis	Positive vs negative	1.30	3.68	<0.001

β* indicates regression coefficient. FIGO, International Federation of Gynecology and Obstetrics. LVSI, Lymph vascular space invasion. DFS, disease free survival.

**Table 3:** Multivariate Cox regression for DFS.

Variables	β*	HR	P
FIGO stage			
IB2		1	
IIA	0.67	1.95	0.06
IIB	0.73	2.07	0.03
Grade			
G1		1	
G2	1.21	3.35	0.048
G3	1.77	5.88	0.004
Undetermined	1.16	3.19	0.07
Cell type			
Squamous		1	
Non-squamous	1.04	2.84	<0.001
Parametrial infiltration			
Negative		1	
Positive	0.83	2.29	<0.001
Lymph node metastasis			
Negative		1	
Positive	1.36	3.89	<0.001

β* indicates regression coefficient. FIGO, International Federation of Gynecology and Obstetrics. DFS, disease free survival Note: β0 = 0.22.

**Table 4:** Three-year disease-free survival rates and 5-year disease-free survival rates among the risk groups.

Variables	3-year DFS rate (%)		5-year DFS rate (%)	
	training	validation	training	validation
Risk groups				
Low risk group	97.7	96.5	97.7	96.5
Intermediate risk group	90.0	84.3	82.3	76.8
High risk group	62.3	60.9	48.7	55.3

DFS, disease free survival.
