# Supplementary material for: Prognostic risk model development and prospective validation among patients with cervical cancer stage IB2 to IIB submitted to neoadjuvant chemotherapy
Source: Sci Rep. 2016 Jun 9;6:27568. doi: 10.1038/srep27568 (PMC4899714; doi:10.1038/srep27568)
Supplement: Supplementary Information [file srep27568-s1.pdf]

Prognostic risk model development and prospective validation among patients with cervical cancer stage IB2 to IIB submitted to neoadjuvant chemotherapy

Kecheng Huang[1,*], Haiying Sun[1,*], Xiong Li[2], Ting Hu[1], Ru Yang[3], ShaoShuai Wang[1], Yao Jia[1], Zhilan Chen[4], Fangxu Tang[1], Jian Shen[2], Xiaomin Qin[5], Hang Zhou[6], Runfeng Yang[7], Juan Gui[8], Lin Wang[1], Xiaolin Zhao[9], Jincheng Zhang[10], Jiong Liu[10], Lili Guo[9], Shuang Li[1,#] & Shixuan Wang[1,#]

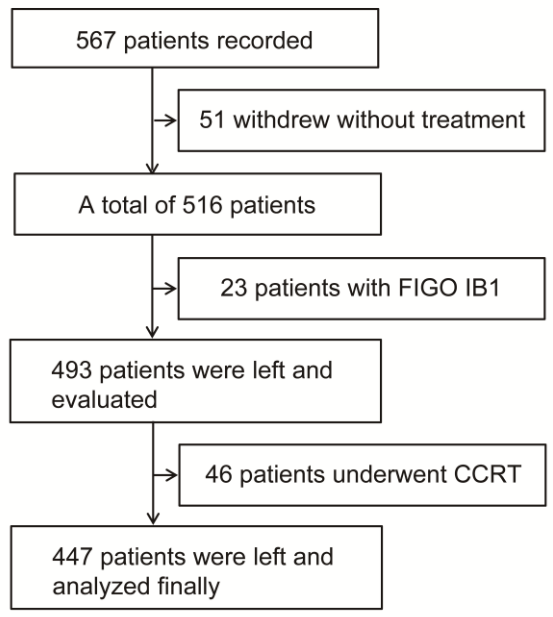

Supplementary Fig. S1. Diagram of patients in the prospective cohort.

Legend: A total of 516 patients were recruited into the study and received treatment; 23 patients with FIGO IB1 were excluded, and 46 patients undergoing concurrent chemoradiotherapy instead of surgery were also excluded; then 447 patients finally underwent surgery after NACT.

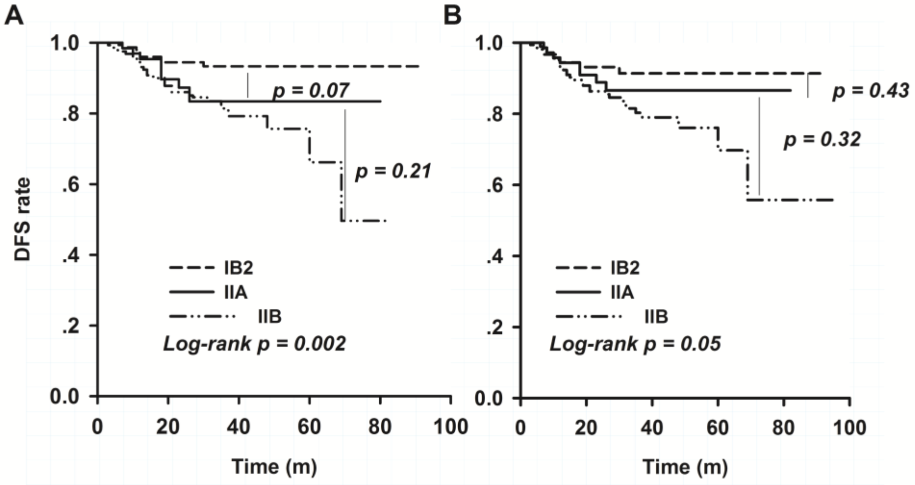

Supplementary Fig. S2. Kaplan-Meier survival estimates of evaluated patients with cervical cancer from both (A) training study and (B) validation cohort.

Legend: Kaplan-Meier survival estimates for low-, intermediate- and high-risk patients with cervical cancer as defined by the FIGO stage system. Disease-specific survival curves of evaluated patients in (A) the training study and (B) the validation cohort. Log-rank test used to calculate *P* values. Statistical significance were observed between the groups.

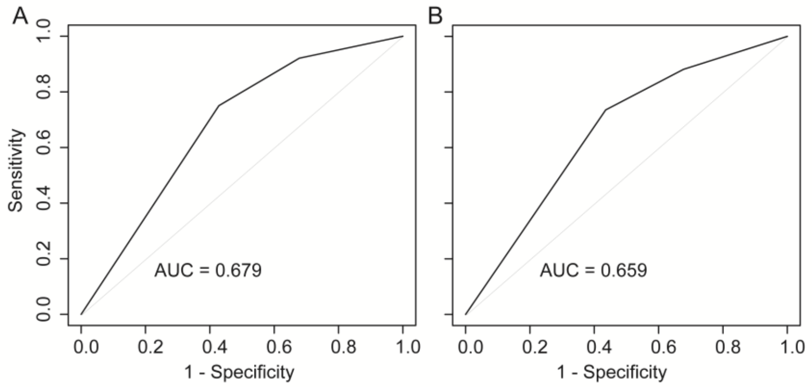

Supplementary Fig. S3. Time-dependent receiver operating characteristic (ROC) curves of evaluated patients with cervical cancer from both (A) training study and (B) validation cohort.

Legend: ROC curves for FIGO stage system were used as predictors of recurrence as result of cervical cancer at the 5th years in (A) the training study and (B) the validation cohort. The area under the ROC curves were < 0.75 in both training study and validation cohort.
